# The apolipoprotein receptor LRP3 compromises APP levels

**DOI:** 10.1186/s13195-021-00921-5

**Published:** 2021-11-02

**Authors:** Inmaculada Cuchillo-Ibañez, Matthew P. Lennol, Sergio Escamilla, Trinidad Mata-Balaguer, Lucía Valverde-Vozmediano, Inmaculada Lopez-Font, Isidro Ferrer, Javier Sáez-Valero

**Affiliations:** 1 Instituto de Neurociencias de Alicante, Universidad Miguel Hernández de Elche-CSIC, Sant Joan d’Alacant, Spain; 2 Centro de Investigación Biomédica en Red sobre Enfermedades Neurodegenerativas (CIBERNED), Madrid, Spain; 3 Instituto de Investigación Sanitaria y Biomédica de Alicante (ISABIAL), Alicante, Spain; 4 Instituto de Neuropatología, Hospital Universitario de Bellvitge, Universidad de Barcelona, Hospitalet de Llobregat, Barcelona, Spain

**Keywords:** sAPP, ApoER2, ApoER2-ICD, Beta-amyloid, Alzheimer’s disease, Chloroquine, Differential centrifugation, Autophagy

## Abstract

**Background:**

Members of the low-density lipoprotein (LDL) receptor family are involved in endocytosis and in transducing signals, but also in amyloid precursor protein (APP) processing and β-amyloid secretion. ApoER2/LRP8 is a member of this family with key roles in synaptic plasticity in the adult brain. ApoER2 is cleaved after the binding of its ligand, the reelin protein, generating an intracellular domain (ApoER2-ICD) that modulates reelin gene transcription itself. We have analyzed whether ApoER2-ICD is able to regulate the expression of other LDL receptors, and we focused on LRP3, the most unknown member of this family. We analyzed LRP3 expression in middle-aged individuals (MA) and in cases with Alzheimer’s disease (AD)-related pathology, and the relation of LRP3 with APP.

**Methods:**

The effects of full-length ApoER2 and ApoER2-ICD overexpression on protein levels, in the presence of recombinant reelin or Aβ42 peptide, were evaluated by microarray, *q*RT-PCRs, and western blots in SH-SY5Y cells. LRP3 expression was analyzed in human frontal cortex extracts from MA subjects (mean age 51.8±4.8 years) and AD-related pathology subjects [Braak neurofibrillary tangle stages I–II, 68.4±8.8 years; III–IV, 80.4 ± 8.8 years; V–VI, 76.5±9.7 years] by *q*RT-PCRs and western blot; LRP3 interaction with other proteins was assessed by immunoprecipitation. In CHO cells overexpressing LRP3, protein levels of full-length APP and fragments were evaluated by western blots. Chloroquine was employed to block the lysosomal/autophagy function.

**Results:**

We have identified that ApoER2 overexpression increases LRP3 expression, also after reelin stimulation of ApoER2 signaling. The same occurred following ApoER2-ICD overexpression. In extracts from subjects with AD-related pathology, the levels of LRP3 mRNA and protein were lower than those in MA subjects. Interestingly, LRP3 transfection in CHO-PS70 cells induced a decrease of full-length APP levels and APP-CTF, particularly in the membrane fraction. In cell supernatants, levels of APP fragments from the amyloidogenic (sAPPα) or non-amyloidogenic (sAPPβ) pathways, as well as Aβ peptides, were drastically reduced with respect to mock-transfected cells. The inhibitor of lysosomal/autophagy function, chloroquine, significantly increased full-length APP, APP-CTF, and sAPPα levels.

**Conclusions:**

ApoER2/reelin signaling regulates LRP3 expression, whose levels are affected in AD; LRP3 is involved in the regulation of APP levels.

**Supplementary Information:**

The online version contains supplementary material available at 10.1186/s13195-021-00921-5.

## Introduction

The members of the family of low-density lipoprotein (LDL) receptors are endocytic receptors that mediate the uptake of lipoproteins and have been classically studied for their role in cholesterol transport and metabolism. Robust evidence indicates that LDL receptor family members are involved in synaptic plasticity regulation and neuronal migration (extensively reviewed in [1–6]). LDL receptors are related to Alzheimer’s disease (AD) pathogenesis as receptors of apolipoprotein E (apoE) [7], being the *APOE4* variant the largest known genetic risk factor for late-onset sporadic AD [8, 9]. Additionally, several members of the LDL receptor family are able to modulate the amyloid precursor (APP) proteolytic processing, either by regulation of the generation of the β-amyloid peptide (Aβ) or through Aβ clearance [10–13].

An important member of the LDL receptor family, ApoER2/LRP8, can exert a modulatory effect in transcriptional expression. ApoER2 interaction with its ligand, the reelin protein, drives to a sequential proteolytic processing, resulting in the cleavage of the receptor by α-secretase, which generates a membrane-tethered C-terminal fragment (ApoER2-CTF), followed by the cleavage by γ-secretase. The action of γ-secretase generates an intracellular domain fragment (ApoER2-ICD) capable of decreasing the expression of reelin mRNA [14, 15]. Using the same brain extracts as in [14], we found later that the generation of ApoER2-CTF appeared lower and, accordingly, reelin expression resulted higher with respect to those in control brain extracts [16].

In this study, we have further explored the modulatory transcriptional activity of ApoER2/reelin signaling, and we have observed that this pathway can modulate the expression of the LDL-related protein 3 (LRP3). LRP3 is probably the most unknown member of a new subfamily of LDL receptors [17], whose precise role in the central nervous system is still undetermined. We have estimated LRP3 expression in the frontal cortex of middle-aged (MA) individuals and in cases with Alzheimer’s disease (AD)-related pathology, and after overexpression in CHO cells. We have demonstrated that LRP3 is able to modulate APP expression.

## Material and methods

### Human brain samples

This study was approved by the ethics committee of Universidad Miguel Hernández de Elche, Spain, and it was carried out in accordance with the WMA Declaration of Helsinki. Brain samples (frontal cortex; see Table 1) were obtained from the Brain Bank of the Institute of Neuropathology, Bellvitge University Hospital. Cases with AD-related pathology were considered those showing neurofibrillary tangles (NFT) and/or senile plaques with the distribution established by Braak and Braak at the post-mortem neuropathological examination [18]. These were categorized as Braak NFT stages I–II *n* = 14, 1 female/13 males, 68.4 ± 8.8 years; Braak stages III–IV, *n* = 14, 7 females/7 males, 80.4 ± 8.2 years; and Braak stages V–VI, *n* = 12, 5 females/7 males, 76.5 ± 9.7 years. Cases at NFT stages I–II showed no or moderate numbers of senile plaques (mostly scores 0 and A); cases at stages III–IV usually had moderate numbers of senile plaques (mostly score B); cases at stages V–VI had heavy senile plaque burden (mostly score C; Table 1). Cases at stages I, II, and III did not have cognitive impairment; three cases at stage IV had moderate cognitive impairment, and cases at stages V and VI had suffered from dementia. Special care was taken not to include cases with combined pathologies to avoid bias in the pathological series. Samples from middle-aged (MA) subjects (3 females/8 males; average age 51.8 ± 4.8 years) corresponded to individuals with no neurological diseases and no evidence of NFTs and senile plaques. The mean post-mortem interval of the tissue was ~8 h in all cases, with no significant difference between the groups.

**Table 1:** Human samples

	Age (y)	Gender	PM (h)	SP	ApoE
**MA NFT**					
0	46	f	9.5	0	ɛ2/ɛ3
	46	m	15		ɛ3/ɛ4
	47	m	5		ɛ3/ɛ3
	49	m	7.5		ɛ3/ɛ3
	50	m	17		ɛ3/ɛ3
	52	m	5		ɛ3/ɛ3
	52	f	6		ɛ4/ɛ4
	53	m	7.5		ɛ3/ɛ3
	56	m	4		ɛ2/ɛ3
	59	m	6,5		ɛ3/ɛ3
	60	f	11.5		ɛ3/ɛ3
**AD NFT**					
Braak I	53	m	6.25	A	ɛ3/ɛ4
	64	m	8.5	0	ɛ3/ɛ3
	67	m	14.5	0	ɛ3/ɛ3
	68	m	11	0	ɛ2/ɛ3
Braak II	57	m	4.5	0	ɛ3/ɛ4
	60	f	9.5	A	ɛ3/ɛ3
	65	m	16.5	0	ɛ3/ɛ3
	67	m	7.25	0	ɛ3/ɛ4
	69	m	3.5	A	ɛ3/ɛ4
	72	m	6.25	A	ɛ3/ɛ4
	74	m	5.5	A	ɛ2/ɛ3
	78	m	16	0	ɛ3/ɛ3
	78	m	10.75	B	ɛ3/ɛ4
	86	m	5.5	A	ɛ2/ɛ3
Braak III	68	f	4.5	A	ɛ3/ɛ3
	71	m	7.5	0	ɛ2/ɛ3
	73	m	4	0	ɛ3/ɛ3
	76	f	4	B	ɛ3/ɛ3
	77	m	13.5	C	ɛ3/ɛ4
	77	m	5.5	A	ɛ3/ɛ3
	79	f	3.5	B	ɛ3/ɛ3
	82	f	5	A	ɛ3/ɛ3
	90	f	4	B	ɛ3/ɛ3
Braak IV	79	m	5	A	ɛ4/ɛ4
	81	f	5	C	ɛ3/ɛ3
	85	m	14	B	ɛ3/ɛ4
	89	m	3.5	B	ɛ3/ɛ4
	99	f	5	B	ɛ3/ɛ3
Braak V	72	m	2.75	C	ɛ3/ɛ4
	73	m	4.5	B	ɛ3/ɛ4
	74	f	9	A	ɛ3/ɛ4
	75	m	11.5	B	ɛ3/ɛ4
	77	m	16	C	ɛ3/ɛ3
	78	m	17	0	ɛ3/ɛ3
	81	f	5.5	C	ɛ3/ɛ4
	87	m	7	C	ɛ3/ɛ3
	93	m	3	C	ɛ3/ɛ3
Braak VI	56	f	7	C	ɛ3/ɛ3
	67	f	8	C	ɛ3/ɛ4
	86	f	20.5	C	ɛ3/ɛ3

Middle-aged (MA) cases and cases with AD-related pathology (AD). Subjects were categorized according to the Braak stage of neurofibrillary tangle (NFT I–VI) and senile plaque staging (0–C) [18, 19]. Age (*y* years), gender (*m* male, *f* female), post-mortem (PM, *h* hours), *SP* senile plaques, *APOE* (*APOE* alleles, ɛ2, ɛ3, and ɛ4)

A major concern in the design of the study is the age of the different groups of human cases. MA individuals are younger (51.8 ± 4.8 years) when compared with cases with AD-related pathology (NFT I–II 68.4 ± 8.8, III–IV 80.4 ± 8.2, and V–VI 76.5 ± 9.7). This selection is due to the fact that the majority of individuals aged 65 years or older have stages I–III of NFT pathology, and, therefore, it is difficult to have samples of age-matched controls without AD-related pathology and morbidities considered in the selection of NFT series that could have an impact on the results [20].

### Cell cultures

SH-SY5Y cells, a human neuroblastoma cell line, were seeded at a density of 1×10^5^ cells/well in 6-well plates and cultured in Dulbecco’s modified Eagle medium (DMEM) supplemented with Glutamax (GIBCO Thermo Fisher Scientific, Rockford, USA), 1% heat-inactivated fetal bovine serum (FBS), penicillin (100 U/ml), and streptomycin (100 μg/ml) in a 5% CO_2_ incubator. To neuro-differentiate the cells, all-trans-retinoic acid (RA, Sigma-Aldrich Co, MO, USA) was employed. RA enhances neuronal markers, reelin and ApoER2 expression [21, 22]. Ten micromolar RA diluted in DMEM with 1% FBS was added every 2 days. After 6 days, cells were treated with recombinant reelin, 12 μg/ml for 24 h. Other cells were treated with suspensions of β-amyloid 1–42 (Aβ_42_) or scrambled control peptide (Aβsc; AIAEGDSHVLKEGAYMEIFDVQGHVFGGKIFRVVDLGSHNVA) (both from Anaspec Peptide, Eurogentec) in DMEM with 1% FBS, for two consecutive days without changing the media, at a final concentration of 500 nM, 1 μM, or 5 μM.

Non-differentiated SH-SY5Y cells were transfected with Lipofectamine 3000 (ThermoFisher) following manufacturer’s instructions, with a construct encoding full-length ApoER2 (pEGFPN1-*Mus musculus* ApoER2, residues 1–842) and ApoER2-ICD-HA expressing only the cytoplasmic domain (residues 728–842) (both generously provided by Dr W. Rebeck; see ref. [23, 24]), or with GFP/cDNA3.1 as mock transfection as in [14] for 48 h. After 24 h post-transfection, some CHO-PS70 cells were treated with 10 μM chloroquine for another 24 h.

CHO cells stably overexpressing wild-type human APP (CHO-PS70, [25]) were grown in DMEM® containing 10% FBS, 0.1% Puromycin (Sigma-Aldrich), and 0.2% G418 disulfate salt (Sigma-Aldrich). CHO-PS70 cells were transfected with full-length human LRP3 cDNA (3×FLAG-LRP3 in pCMV7.1; a kind gift from Christine Lavoie, [26]) for 48 h. After 24 h post-transfection, some CHO-PS70 cells were treated with 10 μM chloroquine for 24 h.

### Brain membrane-enriched fractions

Brain cortex samples were homogenized using a polytron Heidolph RZR-1 at 600–800 rpm, in a glass potter applying 10–15 pulses in buffer at 10% (w/v) (Hepes 1mM, sucrose 0,32 M, Cl_2_Mg mM, EDTA 1mM, NaHCO_3_ 1mM, PMSF, protease inhibitors (Cocktail Complete EDTA free, Roche), antiphosphatase inhibitor (PhosSTOP, Sigma)). The homogenate was centrifuged at 1000 ×*g* during 20 min at 4°C. The supernatant (post-nuclear fraction) was centrifuged at 13000 ×*g* during 15 min at 4°C. The supernatant (cytosolic fraction) was aliquoted, and the resulting pellet (membrane-enriched fraction) was resuspended in buffer (Hepes 1mM, Cl_2_Mg mM, EDTA 1mM, NaHCO_3_ 1mM, PMSF, protease inhibitor cocktail (Sigma-Aldrich), antiphosphatase inhibitor (Sigma-Aldrich)).

In some CHO-PS70 cells, we performed a differential centrifugation. After homogenization of cell extracts in sucrose buffer (0.32 M sucrose, 10 mM Tris pH 7.4, EGTA, 1 mM Na_3_VO_4_, 5 mM NaF, 1 mM EDTA, 1 mM Hepes), the homogenate was centrifuged at 1000 ×*g* for 10 min. The supernatant was centrifuged at 15000 ×*g* for 15 min. The resultant supernatant (fraction containing mainly the plasma membrane and soluble proteins from the cytosol) and the pellet (containing mainly membranes from the endoplasmic reticulum, mitochondria, lysosomes, peroxisomes, and endosomes) were quantified and stored.

### Microarray analysis

Gene expression was analyzed 48 h after transfection with human full-length ApoER2, using microarrays SurePrint G3 Human Microarrays (ID 039494, Agilent Technologies, Spain) and performed by Bioarray SL (http://www.bioarray.es). The concentration and purity of the total RNA extracted were measured by a NanoDrop spectrophotometer, and RNA quality was determined with the kit R6K Screen Tape (Agilent Technologies, Spain). The estimated RNA integrity number ranged between 9.5 and 9.7. Each sample (four samples and four controls) was labeled with Cy3 using the One-Color Microarray-Based Gene Expression Microarrays Analysis v.6.6 (Agilent Technologies, Spain). Data were imported to the linear models for microarray data Bioconductor software (Limma, Marray, affy, pcaMethods and EMA). Raw data were first subjected to background subtraction, then to within-array loess normalization. Finally, across-array normalization was performed. Normalized data were fitted to a linear model. The significance of the gene expression changes was analyzed according to the adjusted *p* value (adj. *p* < 0.05).

### qRT-PCR analysis

RNA was extracted from human brains, SH-SY5Y cells, or CHO-PS70 cells using the TRIzol® Reagent in the PureLink™ Micro-to-Midi Total RNA Purification System (Life Technologies, Carlsbad, CA, USA) following the manufacturer’s instructions. SuperScript™ III Reverse Transcriptase (Life Technologies, Carlsbad, CA, USA) was used to synthesize cDNAs from this total RNA (2 μg) using random primers according to the manufacturer’s instructions. Quantitative PCR amplification was performed on a StepOne™ Real-Time PCR System (Applied Biosystems, Thermo Fisher Scientific, Rockford, USA) with TaqMan probes specific for human *LRP3* (assay ID: HS01041220_m1), *LDLR* (assay ID: HS00181192_m1) (Applied Biosystems, Thermo Fisher Scientific, Rockford, USA), and human *18S* as a housekeeping gene (Applied Biosystems, Thermo Fisher Scientific, Rockford, USA) for the human brain and SH-SY5Y cell samples. In CHO-PS70, mRNA expression was measured with primers for human APP (forward: AACCAGTGACCATCCAGAAC; reverse: ACTTGTCAGGAACGAGAAGG) and for glyceraldehyde 3-phosphate dehydrogenase (GAPDH, forward: AGAAGGTGGTGAAGCAGGCAT; reverse: AGGTCCACCACTCTGTTGCTGT) to normalize the expression levels of the target gene by the ΔCt method curves.

*APOE* genotyping was performed by *q*RT-PCR according to a previously described method [27].

### Recombinant reelin

HEK-293T cells stably transfected with full-length mouse reelin clone pCrl or GFP (mock) (kindly provided by Dr. E. Soriano, Department of Cell Biology, University of Barcelona, Barcelona, Spain) were seeded in 175-cm^2^ flasks at a density of 10×10^6^ cells/flask. After 3 days in culture in Optimem, the supernatants were filtered through 0.2-μm pores and concentrated with an Amicon Ultra 100-kDa size exclusion filter (Merk Millipore, Darmstadt, Germany). For quantification, a coomasie gel was loaded with different volumes of the concentrated supernatants as well as with different bovine serum albumin solutions to perform an extrapolation.

### Western blotting

Brain membrane-enriched fractions, SH-SY5Y extracts, or CHO-PS70 extracts (30 μg) were run on SDS-PAGE (7.5%, 12%, precast 4–15% gradient, or Tris-tricine 16%) after boiling at 98°C for 5 min in 6× Laemmli sample buffer. Proteins were transferred by electrophoresis to nitrocellulose membranes and detected with antibodies against the C-terminal of LRP3 (mouse, 1:100, Sigma-Aldrich, St. Louis, MO, USA), N-terminal of LRP3 (rabbit, 1:100, Sigma-Aldrich), Flag (mouse, 1:1000, Sigma-Aldrich), C-terminal of LDLR (rabbit, 1:200, Sigma-Aldrich), C-terminal of ApoER2 (rabbit, 1: 2000, Abcam, Cambridge, UK), C-terminal of APP (rabbit, 1: 2000, Sigma-Aldrich), N-terminal of APP (rabbit, 1: 2000, Sigma-Aldrich), sAPPα (mouse, 1:1000; IBL, Hamburg, Germany), sAPPβ (rabbit 1:1000; IBL), LC3B (rabbit, 1:2000; Abcam), or α-tubulin (1:4000, Sigma-Aldrich) as a loading control. Primary antibody binding was visualized with fluorescent secondary antibodies (IRDye, 1: 10000), and images were acquired using an Odyssey CLx Infrared Imaging system (LI-COR Biosciences GmbH). Representative whole blots are shown as Supp Fig. 1.

### Immunoprecipitation

Brain extracts (100 μL) or CHO-PS70 extracts (50 μL) were incubated on a roller for 2.5 h at room temperature with 100 μL of magnetic beads (Dynabeads, Merck Millipore) coupled to the C-terminal LRP3 (mouse, Sigma-Aldrich) for brain extracts, C-terminal APP (rabbit, Biolegend) for CHO-PS170 extracts, or mouse/rabbit IgG (negative controls). The input, bound, and unbound fractions were analyzed by western blotting using specific antibodies.

### Immunofluorescence

CHO-PS70 cells overexpressing LRP3-flag were washed with cold Hank-buffered salt solution and fixed with 4% paraformaldehyde and 0.1 M EGTA for 10 min. To stain the plasma membrane, cells were incubated with WGA-FITC (WGA: lectin from *Triticum vulgaris*, FITC (fluorescein) conjugate, Sigma-Aldrich) for 15 min at room temperature, and the nonspecific sites were blocked with 10% (w/v) bovine serum albumin for 30 min. No permeabilization steps were included before or during the incubation with the primary antibodies. Cells were incubated with a primary antibody against Flag (1:200; mouse; Sigma-Aldrich) for 1 h, followed by the secondary antibody (1:200, Cy5 anti-mouse; GE-Healthcare) for 1 h. After washes with PBS, cells were incubated briefly with Hoechst dye to label nuclei (Invitrogen). Pictures were acquired in a Leica SPEII upright TCL-SL confocal microscope using an oil-immersion 40× objective

### Double-labeling immunofluorescence and confocal microscopy

The frontal cortex and hippocampus of 14 cases at Braak NFT stages 0–I, IV, and V–VI and senile plaque stages 0–C were used in the study. Formalin-fixed, paraffin-embedded, de-waxed sections, 4 μm thick, were stained with a saturated solution of Sudan black B (Merck) for 15 min to block autofluorescence of lipofuscin granules present in cell bodies and then rinsed in 70% ethanol and washed in distilled water. The sections were boiled in citrate buffer to enhance antigenicity and blocked for 30 min at room temperature with 10% fetal bovine serum diluted in PBS. Then, the sections were incubated at 4°C overnight with combinations of primary antibodies: LRP3-C-term (Sigma-Aldrich, ref SAB1300316, polyclonal rabbit, diluted at 1:50) and apoER2 (Invitrogen, ref MA5-36130, mouse monoclonal, diluted 1:50). After washing, the sections were incubated with Alexa488 or Alexa546 (1:400, Molecular Probes) fluorescent secondary antibodies against the corresponding host species. Nuclei were stained with DRAQ5^TM^ (1:2000, Biostatus). After washing, the sections were mounted in an Immuno-Fluore mounting medium (ICN Biomedicals), sealed, and dried overnight. Sections were examined with a Leica TCS-SL confocal microscope.

### Statistical analysis

The distribution of data was tested for normality using a D’Agostino-Pearson test. ANOVA was used for parametric variables and the Kruskal-Wallis test for non-parametric variables for comparison between groups. A Student’s *t*-test for parametric variables and a Mann-Whitney *U* test for non-parametric variables were employed for comparison between two groups and for determining *p* values. For data analyzed using unpaired Student’s *t*-test, a Welch’s correction was employed in data with different standard deviations. Correlation between variables was assessed by linear regression analyses. The results are presented as the means ± SE, and all the analyses were performed using GraphPad Prism (version 7; GraphPad Software, Inc). *p* value < 0.05 was considered significant.

## Results

### ApoER2 overexpression increases the expression of LRP3

**Fig. 1:**
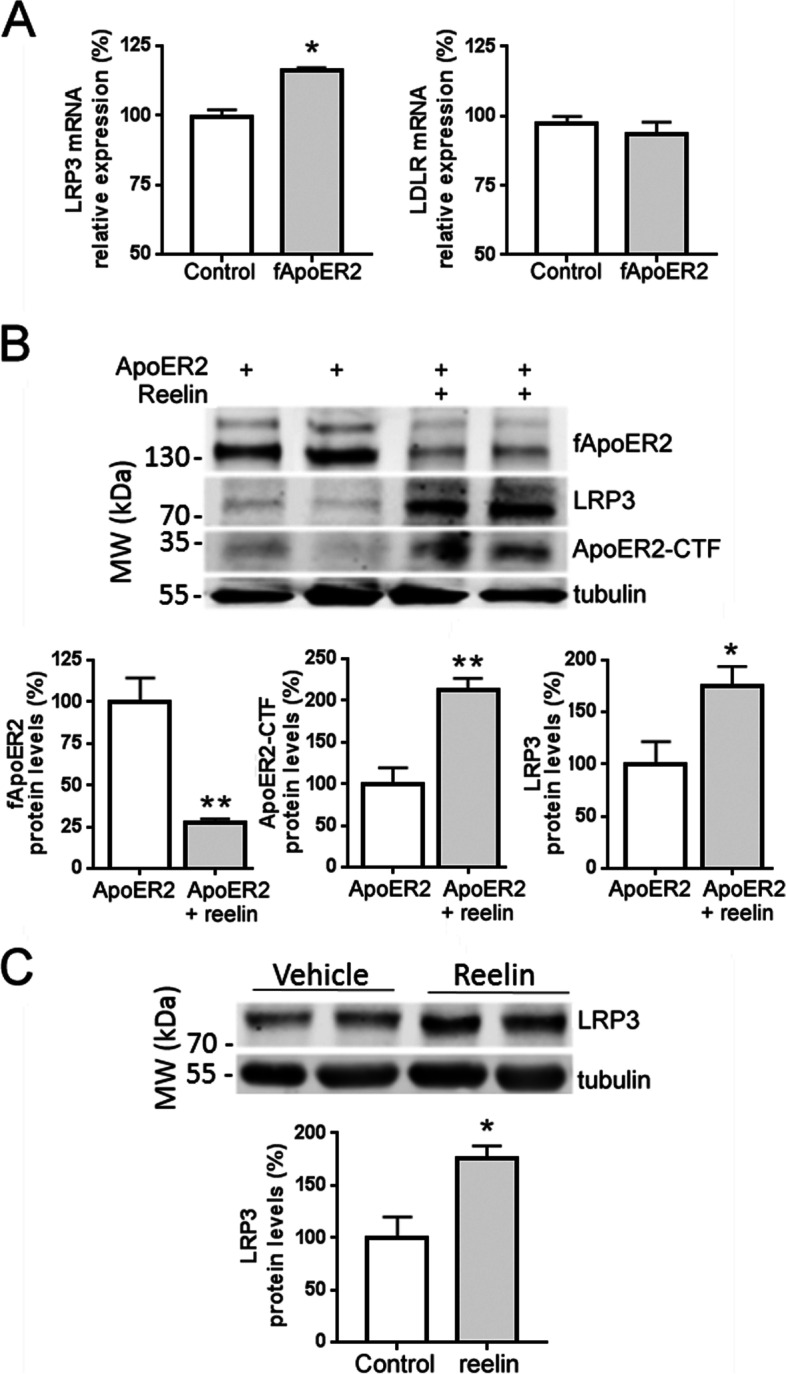
ApoER2/reelin signaling upregulates LRP3 expression. **a** qRT-PCR analysis showing expression of LRP3 mRNA and LDLR mRNA after transfection with GFP cDNA (control) and full-length ApoER2 cDNA (fApoER2) in SH-SYSH cells. 18S was used as an internal control for mRNA expression (*n* = 10–12 for each condition, *p* < 0.001 for control versus fApoER2; *t*-test with Welch’s correction). Note that the *X* axis begins at 50%. **b** Quantification and western blot showing the expression of full-length ApoER2, ApoER2-CTF, and LRP3 proteins after fApoER2 transfection and reelin (12 μg/ml) treatment for 24 h in SH-SY5Y cells. Tubulin was used as an internal control (*n* = 9 for each condition, **p < 0.001 for expression of fApoER2, *t*-test with Welch’s correction, and ApoER2-CTF, *t*-test; *p < 0.05 for expression of LRP3, *t*-test). **c** Quantification and western blot showing the expression of LRP3 protein after reelin (12 μg/ml) treatment for 24 h or vehicle (Hanks’s media) in neuro-differentiated SH-SY5Y cells with retinoic acid (*n* = 9 for each condition, *p <0.05 t-test)

SH-SY5Y cells were transfected with full-length ApoER2, and after 48 h, a microarray was performed. Among the genes affected, we focused on the analysis of LDL receptors and apolipoprotein-related genes (Table 2). The receptors LRP3 and LDLR appeared significantly upregulated, both of which are members of the LDL receptor family. Upregulation of LRP3 was confirmed by *q*RT-PCR, with a significant increase in mRNA LRP3 level compared to its expression in non-transfected cells. However, increments in LDLR mRNA expression were not significant when assessed by *q*RT-PCR (Fig. 1a).

**Table 2:** Expression of genes upregulated by full-length ApoER2 overexpression

Symbol	Gene name	Genomic location	Function		logFC	adj ***p***
**LRP3**	Low-density lipoprotein receptor-related protein 3	19q13.11	Internalization of lipophilic molecules and/or signal transduction Precise role is unclear		0.48	0.047
**LDLR**	Low-density lipoprotein receptor	19p13.2	Mediates endocytosis of cholesterol-rich LDL		0.43	0.018
APOL1	apolipoprotein L, 1	22q12.3	Minor apoprotein component of HDL		1.28	0.003
INSIG1	Insulin-induced gene 1	7q36.3	Regulation of cholesterol cell concentration		0.72	0.001
DHCR24	24-Dehydrocholesterol reductase	1p32.3	Cholesterol metabolic process		0.35	0.008
MVK	Mevalonate kinase	12q24.11	Cholesterol metabolic process		0.33	0.019

Genes associated with lipid binding and transport, and cholesterol metabolism, whose transcripts were upregulated in ApoER2 overexpressing SH-SY5Y cells compared with control cells transfected with an empty vector. The expression of the genes was analyzed on DNA microarrays. The fold change (logFC) in gene expression between samples and controls, as well the adj *p* (*p* value adjusted for multiple testing) is indicated

Although SH-SY5Y cells secrete reelin to the media and it can act in a paracrine mode, recombinant reelin was employed to treat overexpressing-ApoER2 cells to potentiate the ApoER2 signaling. This treatment induced ApoER2 cleavage and, consequently, reduced the amount of full-length ApoER2 and increased the generation of the ApoER2-CTF. Importantly, reelin treatment induced an increment of LRP3 protein levels (Fig. 1b). In RA neuro-differentiated SH-SY5Y cells, reelin treatment was also able to induce an increase in LRP3 protein levels compared to non-stimulated cells (Fig. 1c).

### Expression of ApoER2-ICD upregulates LRP3 expression

**Fig. 2:**
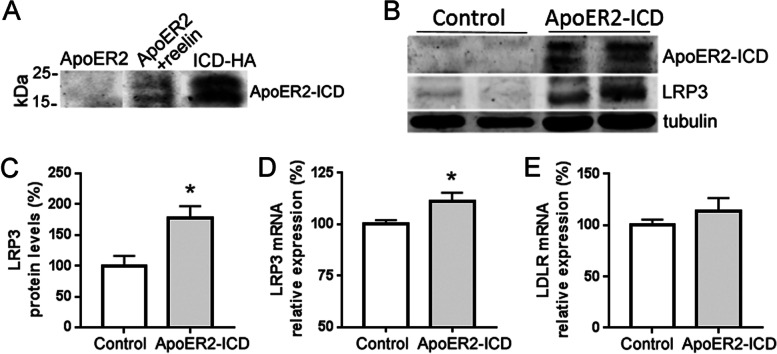
ApoER2-ICD increases LRP3 expression. **a** Representative western blot showing the expression of ApoER2-ICD after transfection with full-length ApoER2 cDNA (ApoER2) and reelin treatment (12μg/ml, ApoER2 + reelin) for 24 h in SH-SY5Y cells. For comparison, the expression of the ApoER2-ICD construct (ApoER2-ICD) is also shown. **b** Western blot and **c** quantification of LRP3 protein expression after ApoER2-ICD transfection in SH-SY5Y cells. Tubulin was used as an internal control (*n* = 6 for each condition, *p < 0.001, *t*-test). **d** qRT-PCR analysis showing the expression of LRP3 mRNA (*n* = 7 for each condition, *p < 0.05, *t*-test) and **e** LDLR mRNA (*n* = 10 for each condition) after transfection with GFP cDNA (control) and ApoER2-ICD cDNA in SH-SY5Y cells. Note that the *X* axis in **d** begins at 50%. 18S was used as an internal control for mRNA expression

We considered the possibility that increments of LRP3 expression were induced by ApoER2-ICD, a fragment with transcriptional regulatory activity [14], generated by the proteolytic cleavage of ApoER2-CTF. This small fragment was observed in ApoER2-overexpressing cells after treatment with reelin (Fig. 2a). Thus, we overexpressed a chimeric ApoER2-ICD (amino acid residues 728–842) and measured LRP3 expression. LRP3 mRNA expression and protein levels increased significantly with respect to non-transfected cells (Fig. 2b–d), while LDLR mRNA levels were not significantly affected by ApoER2-ICD (Fig. 2e).

### Expression levels of LRP3 in Aβ42-treated cells

**Fig. 3:**
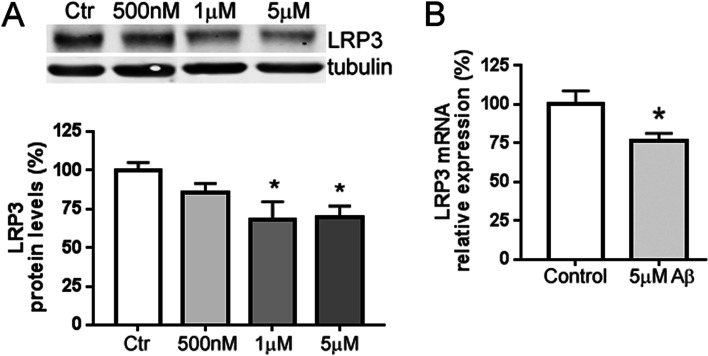
Aβ42 reduces LRP3 expression. **a** Quantification and western blot showing the expression of LRP3 proteins in neuro-differentiated SH-SY5Y cells treated with 5 μM Aβ42 or scrambled Aβ42 (control). Tubulin was used as an internal control (*n* = 9 for each condition, *p < 0.05, *t*-test). **b** qRT-PCR analysis showing expression of LRP3 mRNA in neuro-differentiated SH-SY5Y cells treated with 500 nM, 1μM, 5 μM Aβ42, or scrambled Aβ42 (control). 18S was used as an internal control for mRNA expression (*n* = 8 for each condition, *p* < 0.05; *t*-test)

On the contrary to the upregulation of LRP3 mRNA and protein that we observed after overexpression of full-length ApoER2 or ApoER2-ICD, we expected to find less LRP3 expression in Aβ42-treated cells, due to the fact that Aβ treatment reduces the generation of ApoER2-CTF [16]. In agreement with this view, we found that treatment of neuro-differentiated SH-SY5Y cells with 1 μM and 5μM Aβ42 decreased the LRP3 protein levels, but 500 nM did not have the same effect, in comparison to scrambled peptide treatment (control, Fig. 3a). Five micromolar Aβ42 also reduced LRP3 mRNA expression (Fig. 3b).

### Expression levels of LRP3 in AD brain

**Fig. 4:**
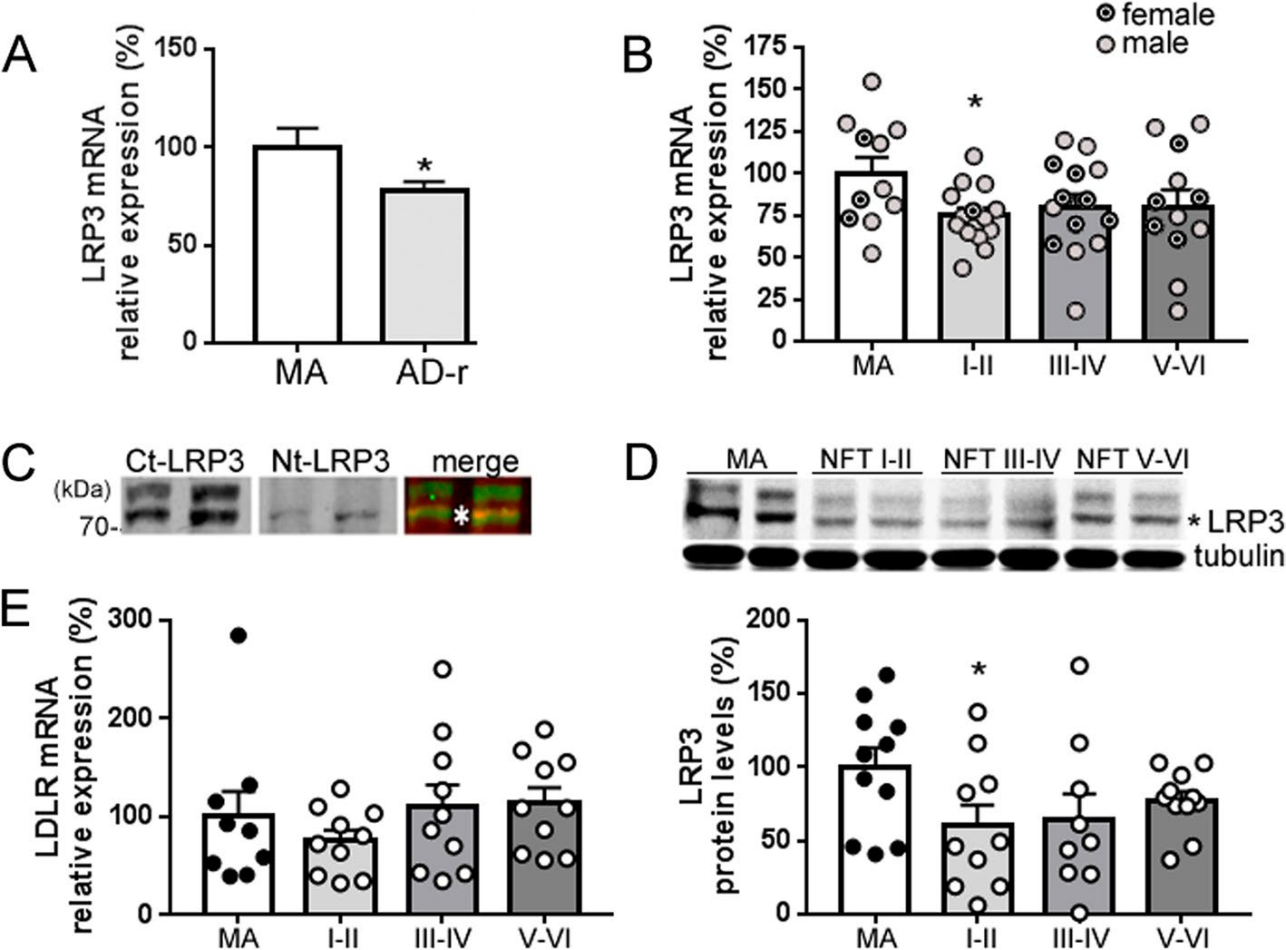
Low levels of LRP3 in AD frontal cortex. **a** qRT-PCR analysis showing expression of LRP3 mRNA in brain extracts from MA and Alzheimer’s disease-related (AD-r) subjects, and **b** categorized by Braak NFT stages (I–II, III–IV, and V–VI). 18S was used as an internal control for mRNA expression (*n* = 11 for MA, *n* = 12–14 for each AD-r Braak stage, *p < 0.05, *t*-test for MA *v* AD-r, *t*-test with Welch’s correction for MA *v* AD-r I–II). **c** Western blots showing different LRP3 immunoreactivities in human cortex extracts. Two bands were observed using an anti-C-terminal LRP3, but a single band was observed when an anti-N-terminal LRP3 was used, all between 70 and 100kDa. One of the bands immunoreacted to both antibodies, likely representing the full-length receptor. Accordingly, the overlapping band (*) was selected for quantification. **d** Western blot using an anti-C-terminal LRP3 in brain extracts from MA and AD-r subjects, categorized by Braak’s stages (NFT I–II, NFT III–IV, and NFT V–VI) and quantification of the lower band (marked with a *). Tubulin was used as an internal control (*n* = 11 for MA, *n* = 10–11 for each NFT Braak’s stage, *p < 0.05, Mann-Whitney test). **e** qRT-PCR analysis showing expression of LDLR mRNA in brain extracts from MA and AD-r subjects, categorized by Braak’s stages. 18s was used as an internal control for mRNA expression (*n* = 9 for MA, *n* = 10 for each Braak’s stage)

Next, we examined LRP3 levels in human frontal cortex extracts. Considering all cases with AD-related pathology, LRP3 mRNA expression was lower with respect to MA subjects (*p* = 0.02; *t*-test) (Fig. 4a). However, when cases with AD-related pathology were categorized by Braak NFT stages, the reduction was significant only at Braak stages NFT I–II (*p* = 0.03; *t*-test), while NFT III–IV or NFT V–VI displayed the same trend but failed to reach statistical significance (*p* = 0.10; *p* = 0.15, respectively, *t*-test). No significant modifications were found between Braak stages NFT I–II and NFT III–IV or NFT V–VI (*p* = 0.56; *p* = 0.65, respectively, *t*-test; Fig. 4b). Despite the difference in age between MA and AD-related pathology cases, age did not correlate with LRP3 mRNA in MA (*n* = 11; *R* = 0.058, *p* = 0.87) or AD-related pathology individuals (*n* = 40; *R* = 0.067; *p* = 0.68).

Gender did not contribute to differences in LRP3 mRNA expression either. The comparison between females and males from MA and from AD-related pathology groups was not statistically significative (*p* = 0.13, one-way ANOVA). When female values were subtracted from both groups, LRP3 mRNA expression in males was still different between MA and AD-related pathology overall (*p* = 0.042, *t*-test). However, the difference observed in Braak stages I–II failed to maintain statistical significance, probably due to the smaller sample size (*p* = 0.060, *t*-test). Braak stages III–IV and V–VI remained without differences in males compared to MA males (*p* = 0.20 and *p* = 0.22, respectively, *t*-test). The *APOE* genotype did not account for LRP3 mRNA expression either (*p* = 0.47 ɛ4 carriers *v* non-ɛ4 carrier AD-related cases).

To evaluate LRP3 protein levels in the cortex from MA and cases with AD-related pathology, membrane-enriched fractions were isolated from brain samples. Due to the lack of reports about LRP3 in the brain, two antibodies were tested to corroborate the identity of LRP3 immunoreactive bands (Fig. 4c). We found that LRP3 expression levels were lower at Braak stages I–II compared to those in MA individuals (*p* = 0.048, *t*-test, Fig. 4d). No further differences were seen at stages III–IV and V–VI when compared with MA (*p* = 0.11 and *p* = 0.12, respectively, *t*-test) and compared with Braak NFT stages I–II (*p* = 0.84 and *p* = 0.26 respectively, *t*-test).

The estimated expression of LDLR mRNA was not significantly different between MA individuals and AD-related pathology subjects when the extracts were compared overall (*p* = 0.73 Mann-Whitney) or when compared discriminating Braak stages (*p* = 0.73 one-way ANOVA; Fig. 4e).

### LRP3 interacts with apoE and APP, but not with reelin in the human brain

**Fig. 5:**
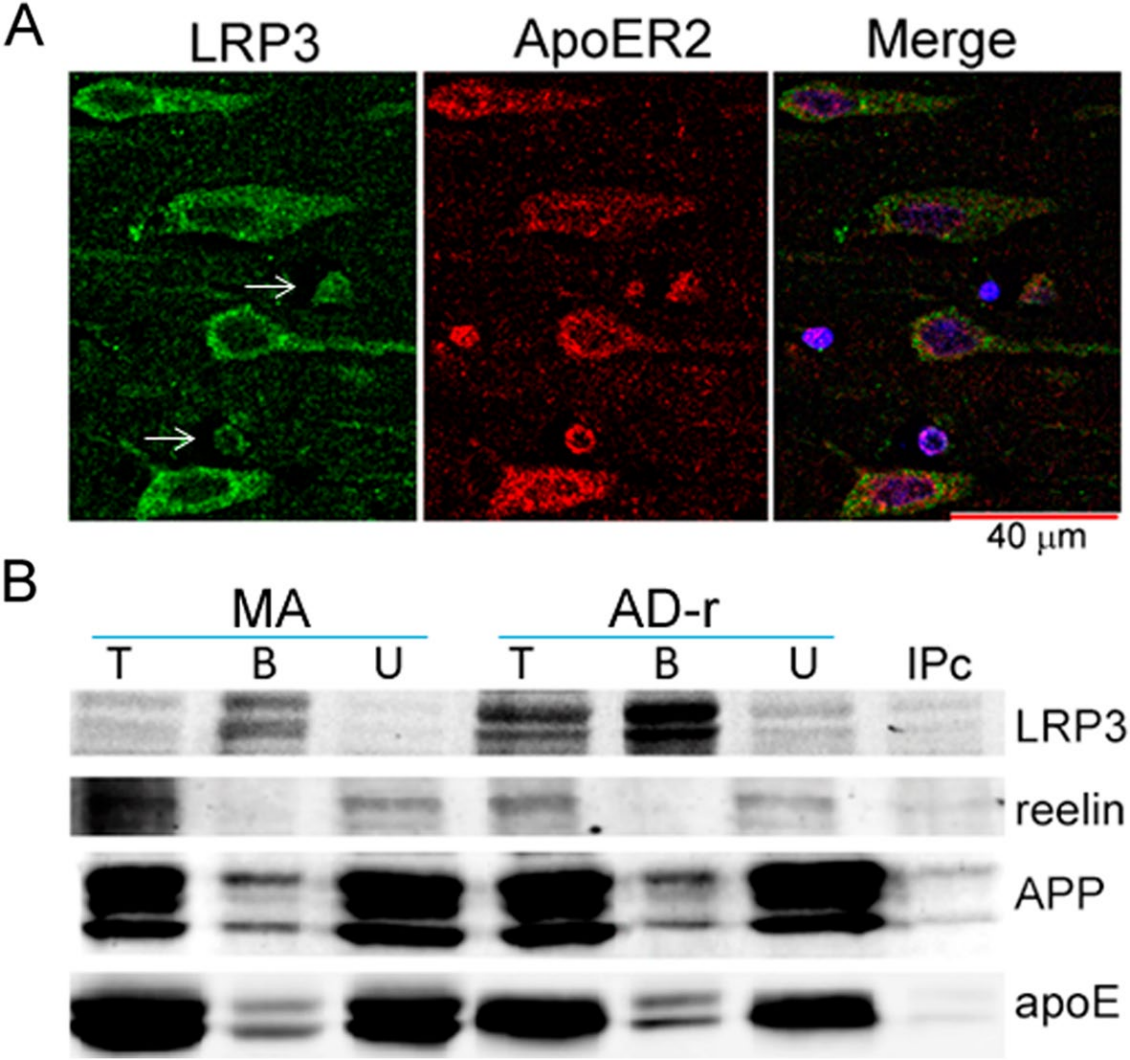
LRP3 co-immunoprecipitates with apoE and APP. **a** Representative immunofluorescence photomicrograph showing LRP3 and ApoER2 labeling in the same cells in a hippocampus slice of a MA subject. Neurons (large cells) show LRP3 and ApoER2 co-localization; in addition, oligodendroglia-like cells (thin arrows) also co-localize both antibodies. **b** Representative western blots showing immunoprecipitation of LRP3 and co-immunoprecipitation of reelin (no immunoprecipitation), apoE, and APP, from non-demented (ND) and Alzheimer’s disease (AD) extracts. T total input, B bound fraction, U unbound fraction, IPc bound fraction of the negative control

Double-labeling immunofluorescence and confocal resolution showed that the LRP3 antibody recognized small granules localized in the cytoplasm and proximal dendrites of all neurons, and around the nucleus of glial cells in the hippocampus and frontal cortex. ApoER2 antibody also showed small granules in the cytoplasm of neurons and small glial cells. The immunostaining was variable in the MA group and in cases with NFT pathology with marked individual disparities, probably due to the vulnerability of the protein to the pre-mortem status and post-mortem delay (Fig. 5a). This individual variability did not permit any attempt to quantify inter-group immunostaining densitometry.

We also evaluated, by means of immunoprecipitation assays, whether reelin acts as a ligand for LRP3, as it does for ApoER2, in frontal cortex extracts from MA and AD-related pathology cases. Reelin was not co-immunoprecipitated from any brain extracts. We next assessed whether LRP3 interacts with apoE and APP, in the same way as many members of the LDL receptor family do. After immunoprecipitation, both proteins were co-immunoprecipitated with LRP3 in MA and cases with AD-related pathology (Fig. 5b).

### LRP3 modulates APP expression levels

**Fig. 6:**
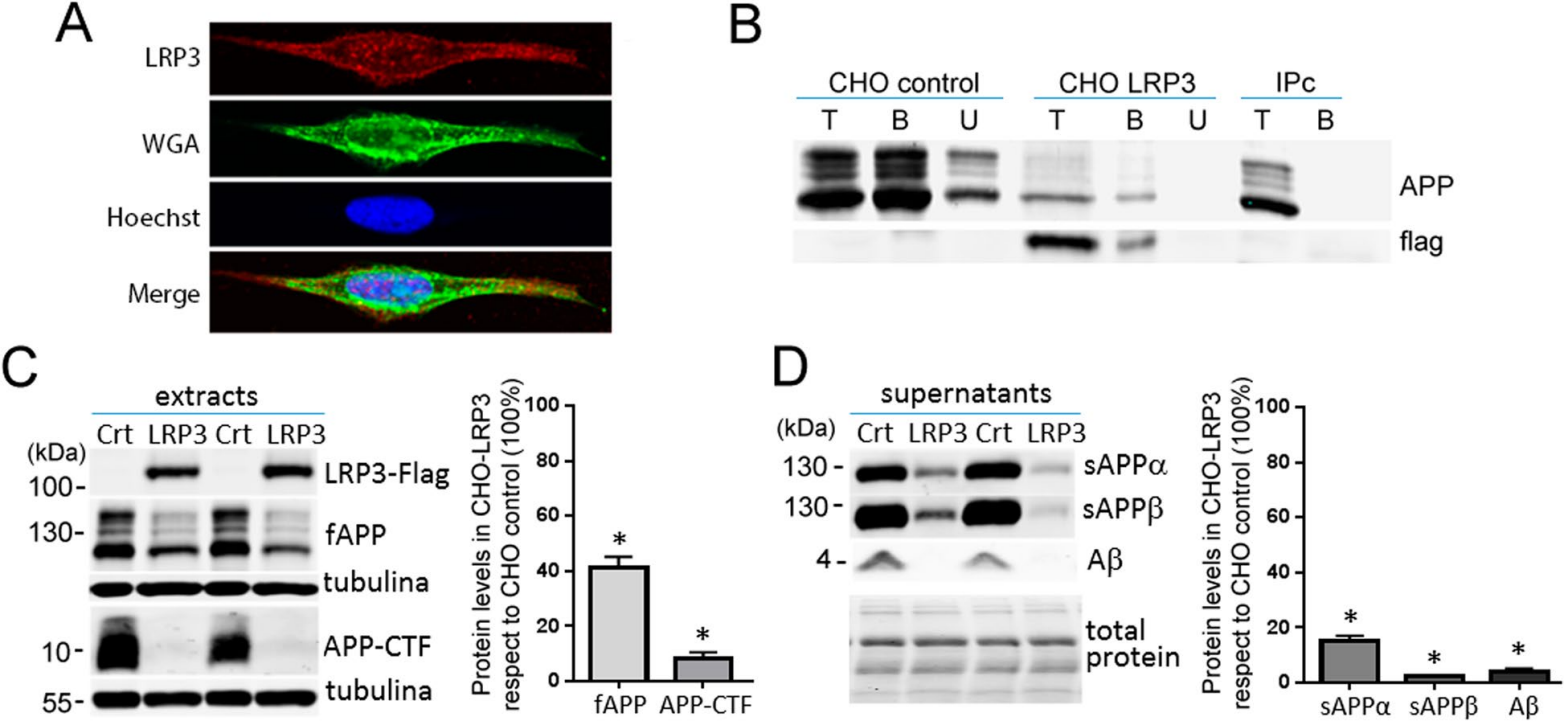
LRP3 overexpression in CHO-PS70 cells decreases APP levels. **a** Representative immunofluorescence photomicrographs showing LRP3 and WGA labeling at the plasma membrane and discrete cytosolic areas of CHO-PS70 cells transfected with LRP3-flag cDNA. Nuclei were stained with the DNA dye Hoechst. **b** Western blots from CHO-PS70 cells transfected with LRP3-flag (CHO-LRP3) or pcDNA3.1 (CHO control) showing immunoprecipitation of APP (using a C-terminal APP antibody) and co-immunoprecipitation of APP (using an N-terminal APP antibody) and LRP3. T input, B bound fraction, U unbound fraction, IPc negative control. **c** qRT-PCR analysis showing expression of APP mRNA in CHO-PS70 transfected with LRP3-flag (LRP3) or pcDNA3.1 (Ctr, control). *GAPDH* was used as an internal control for mRNA expression (*n* = 13 for each condition). **d** Western blots and quantification of CHO-PS70 cells transfected with LRP3-flag (LRP3) or pcDNA3.1 (Ctr, CHO control) showing the expression in the cell extracts of LRP3, full-length APP, APP-CTF proteins, and tubulin, as an internal control (*n* = 6 for each condition, *p<0.001, *t*-test). **d** Western blots and quantification of CHO-PS70 cells transfected with LRP3-flag (LRP3) or pcDNA3.1 (Ctr, CHO control) showing the expression in the supernatants of sAPPα, sAPPβ, and Aβ, and the total protein as the internal control (*n* = 6 for each condition, *p<0.001, *t*-test)

**Fig. 7:**
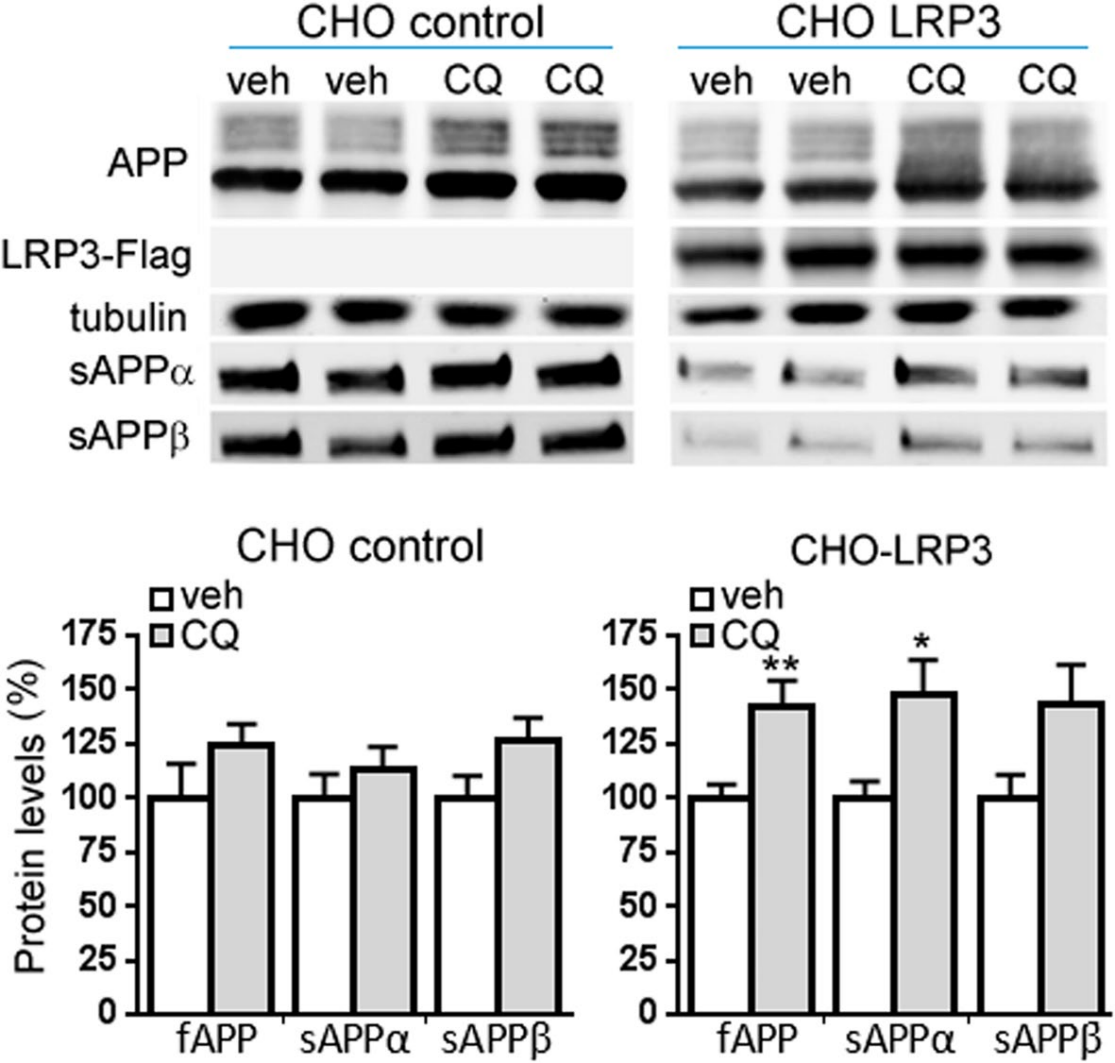
Inhibition of lysosomal/autophagy function increases APP and sAPP levels. Western blots and quantification of CHO-PS70 cells transfected with LRP3-flag (CHO LRP3) or pcDNA3.1 (CHO) for 24h and then treated with 10 mM chloroquine (CQ) for another 24 h or with vehicle (veh, Hank’s media). Western blots show the expression in the cell extracts of LRP3, full-length APP (fAPP), and tubulin, and the expression of sAPPα and sAPPβ from the supernatants (*n* = 6–14 for each condition, **p<0.01 for fAPP respect to control; **p<0.05 for sAPPα respect to control; *p* = 0.065 for sAPPβ)

We tested whether LRP3 was able to influence APP processing and Aβ generation in a similar manner to other members of the LDL receptor family. In order to do so, we overexpressed LRP3 in CHO-PS70 cells, a cell line that expresses the wild-type APP770 isoform. LRP3 was located at discrete areas of the soma and in the plasma membrane of CHO-PS70 cells (Fig. 6a). Moreover, LRP3 and APP co-immunoprecipitated in these cells (Fig. 6b). Overexpression of LRP3 did not affect APP mRNA levels (Fig. 6c), but it drastically reduced full-length APP levels, as well as APP-CTF in cell extracts (Fig. 6d). In the supernatant, the levels of sAPPα, sAPPβ, and soluble Aβ decreased in transfected CHO-PS70 cells compared to mock-transfected cells (Fig. 6e). Interestingly, when lysosomal function was impaired by chloroquine, full-length APP and sAPPα levels increased in a significant manner with regard to non-treated cells (*p* = 0.0044; *p* = 0.031, respectively, *t*-test; Fig. 7). sAPPβ levels showed a tendency to be higher than non-treated cells (*p* = 0.065).

**Fig. 8:**
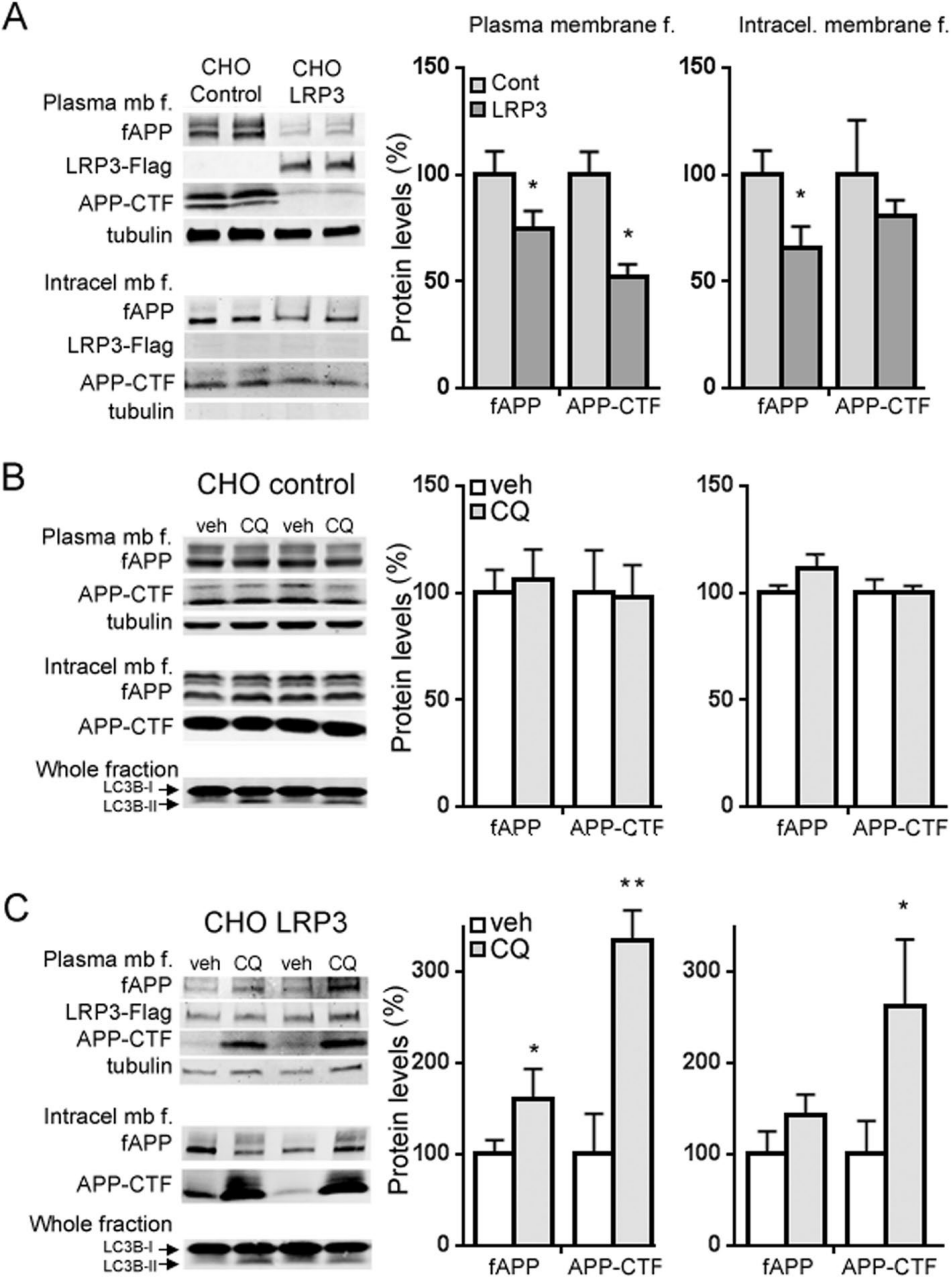
Chloroquine increases full-length APP and sAPP levels at the membrane fraction. Western blots and quantification of the expression of full-length APP (fAPP), APP-CTF, Flag (LRP3-flag), and tubulin in cytosol and plasma membrane-containing fractions (plasma membrane f. or plasma mb f.) and in intracellular membrane-containing fractions (intracell membrane f. or intracell mb f.) from CHO-PS70 cell homogenates. The presence of LRP3-flag was only observed in the cytosol and plasma membrane fraction, and tubulin was not present in the intracellular membrane-containing fraction. For LC3B-I conversion to LC3B-II to monitor autophagy, the whole cell extracts were used. **a** Comparison between CHO-PS70 cells transfected with LRP3-flag (CHO LRP3) or pcDNA3.1 (CHO control). **b** Comparison between CHO cells transfected with pcDNA3.1 (CHO control) for 24h and then treated with 10 mM chloroquine (CQ) for another 24 h or with vehicle (veh, Hank’s media). **c** Comparison between CHO cells transfected with LRP3-flag (CHO LRP3) for 24h and then treated with 10 mM chloroquine (CQ) for another 24 h or with vehicle (veh, Hank’s media). (*n* = 6–10 for each condition, **p<0.01; *p<0.05 *t*-test)

To determine in more detail whether LRP3 is involved in APP degradation by lysosomes, we performed a differential centrifugation of CHO-PS70 cell homogenates. Two different fractions were obtained: a cytosol and plasma membrane-containing fraction, and an intracellular membrane-containing fraction. In CHO cells overexpressing LRP3, full-length APP levels were lower in both fractions, but APP-CTF levels were lower only in the intracellular membrane-containing fractions compared to those in CHO controls (Fig. 8a). Treatment with chloroquine did not affect APP levels in CHO cell controls in any fraction (Fig. 8b). In CHO cells overexpressing LRP3, full-length APP and APP-CTF levels increased in the cytosol and plasma membrane-containing fractions after chloroquine treatment. This could indicate that chloroquine is affecting LRP3 capacity of inducing APP endocytosis from the plasma membrane as observed in Fig. 8a. However, only APP-CTF levels were higher than those in CHO controls in the intracellular membrane-containing fractions (Fig. 8c). This could indicate an accumulation of APP-CTF in vesicles such as endosomes or autophagosomes, whose fusion with lysosomes is inhibited by chloroquine.

## Discussion

Our results suggest that reelin signaling, through the cleavage of its receptor ApoER2, can ultimately influence the expression of other liporeceptors, such as LRP3. Many LDL receptor family members, such as ApoER2, LDLR, LRP1, LRP1b, LRP6, and SorLA (LRP11), as well as other alternative apoE receptors such as Trem2, are γ-secretase substrates [28, 29]. For many of these receptors, the nuclear translocation of the respective ICDs and their transcriptional functions have been demonstrated or inferred [13, 30–32]. Here, we demonstrate that reelin-induced generation of ApoER2-ICD, as well as ApoER2-ICD overexpression, increases LRP3 expression. This supports a link between ApoER2 processing and the regulation of the alternative apoE liporeceptor LRP3.

In frontal cortex extracts from AD, where ApoER2/reelin signaling is impaired and ApoER2 processing is lessened (reviewed in [33]), we found lower LRP3 protein and mRNA levels. LRP3 expression was mainly affected at early Braak stages of NFT pathology (stages I–II), in which the trans-entorhinal region shows neurofibrillary tangles and neuropil threads [18]. However, since the same decreasing trend was determined in advanced Braak stages, additional studies are needed to determine whether LRP3 decrease is only an early phenomenon associated to AD-related progression.

In the microarray, after overexpression of full-length ApoER2, the expression of another LDL receptor family member, LDLR, also appears to be upregulated. Interestingly, both LRP3 and LDLR are encoded by genes located on chromosome 19, locus 19q13 [34, 35]. The *APOE* gene also maps in chromosome 19, on locus 19q13.32 [36], in a cluster together with the apolipoprotein C1 and C2 genes. Genetic linkage studies suggest the presence of AD risk genes on chromosome 19 that would act in an independent manner from apoE, such as *ABCA7* (19p13.3) and *CD33* (19q13.41) [37]. Indeed, *LDLR* was analyzed as a potential AD risk factor, but the study concluded that the genetic variants in LDLR did not make a significant contribution to AD risk in the general population [38]. Interestingly, recent multiplex proteomics studies have identified that LDLR levels are modestly decreased in CSF from early AD patients, suggesting that this receptor could represent a new specific biomarker for AD [39]. Other genes encoding LDL receptor family members, such as LRP1, LRP1b, LRP2, LRP4, LRP6, and SorLA, have been associated to AD risk (reviewed in [13]), as well as ApoER2 [40]. Despite the results from the microarray study, the *q*RT-PCR failed to corroborate the modulation of LDLR by ApoER2 and did not find changes on LDLR expression in AD extracts.

The reelin receptors ApoER2 and VLDLR are core members of the LDL family that share the same extracellular domain structure, the ligand binding-type repeat domains (LBDs) and the EGF-precursor homology domains. The intracellular domain of each of the core members contains at least one NPxY (Asn-Pro-X-Tyr) motif, which plays a role in protein interaction/signal transduction [41–43] and endocytosis [44]. In comparison, LRP3 is smaller than the core members of the LDL receptor family. LRP3 belongs to a subfamily, together with LRP10 (murine LRP9), LRP12, and Lrad3 (ST7/Mig13). These subfamily members are characterized by the sole presence of LBDs and CUB-domains (which binds Complement, Uegf, and Bmp1) in their extracellular domain and lack the EGF-like repeats [13]. The short LBD in LRP3 is likely the domain responsible for the co-immunoprecipitation of apoE, as this is the competent region that binds several ligands [45]. However, reelin did not co-immunoprecipitate, in the same manner as receptor-associated protein (RAP), another ApoER2 ligand, which does not bind to LRP3 either [17, 34, 45, 46]. In the intracellular domain, LRP3 lacks the NPxY motifs, but instead contains a similar tyrosine-based sequence (EDFPVY) [34, 47]. Therefore, the domain by which APP is able to interact with LRP3 is yet to be determined. In vitro data showed that the extracellular domain of LRP10 interacts with APP [48], while Lrad3, the LDL receptor family member with the shortest extracellular domain [49], is also able to interact with APP and to modulate APP processing pathways. ApoER2 and APP are linked extracellularly by binding different domains of F-spondin [50] and intracellularly through the adaptor proteins Dab-1 and Fe65, which interact with the NPxY motif of ApoER2 and APP [24, 51, 52]. Therefore, more studies are needed to explore the direct or indirect interaction between LRP3 and APP.

We observed that overexpression of LRP3 decreased the levels of full-length APP and APP-CTF in the fraction containing the plasma membrane, as well as Aβ and soluble APP fragment levels generated after amyloidogenic and non-amyloidogenic processing pathways. In CHO-PS70 cells overexpressing LRP3, chloroquine treatment increased the levels of full-length APP and APP-CTF in the fraction containing the plasma membrane, and of sAPPα in the media; furthermore, APP-CTF levels in the fraction containing intracellular vesicles were higher when autophagy was inhibited compared to non-treated cells. This suggests that LRP3, described as an endocytosis receptor [34], could be involved in APP processing through lysosomal degradation/autophagy mechanisms. The blockage of LRP3-mediated APP internalization by chloroquine could explain the increase in sAPPα levels, but not sAPPβ, as it has been proposed that the cleavage of APP by α-secretase occurs mainly at the cell surface [53], and also the increase of APP-CTF in intracellular vesicles, as endosomes would not be able to fuse with autophagosomes, thus leading to the accumulation of APP-CTF. Core members of the LDL receptor family have also been associated with APP trafficking and internalization, thus determining APP proteolytic processing and Aβ production, which could play a role in AD pathogenesis [54–57]. For example, LRP1 increases APP endocytosis and generation of Aβ [58–60], while LRP1B retains APP at the cell surface [61]. ApoER2 is able to alter APP subcellular distribution, increasing the generation of Aβ; this effect depends on the integrity of the NPxY motif in ApoER2 [62]. In a mouse model in which the ApoER2 isoform lacks three LBDs, the non-amyloidogenic processing of APP predominates [63]. In this line, LRP1 endocytosis impairment favors non-amyloidogenic processing of APP due to reduced internalization, resulting in less extracellular Aβ [64, 65]. Additionally, mechanisms related to the APP secretory pathways are also possible, such as for LRP1, whose retention in the endoplasmic reticulum by the expression of a specific motif leads to a decrease in full-length APP and CTF levels at the plasma membrane as well as in Aβ secretion [1, 66]. A direct downregulation of APP mRNA would be unlikely given our *q*RT-PCR data.

Interestingly, LRP1 has been shown to constitute a major regulator of tau uptake and spread [67]. Therefore, the potential tau-LRP3 interactions appear to be an interesting possibility to study. A thorough investigation of possible interactions of LRP3 with AD hallmarks and key proteins could serve to decipher the physiological role and potential participation in pathological processes of this LDL receptor family member.

LRP3 expression is highest in skeletal muscle and in the ovaries, but it is also present at relatively high levels in the brain and heart, among other tissues [17]. LRP3 has been involved so far in osteogenic and adipocytic differentiation [68], and systemic use of steroids has been associated with site-specific differential methylation of the LRP3 gene [69], but its role in neuronal activity is still unknown. *LRP*3 has been identified as a gene upregulated for a short window of 2 h, exclusively following learning, in the rat dentate gyrus [70]. To clarify LRP3s biological functions, it is essential to define the significance of LRP3 expression in the brain in aging and AD-related pathology with disease progression. An alteration in the expression of LRP3 may influence the processing and expression of APP, affecting its synaptic function and, therefore, contributing to the AD pathology.

## Conclusions

ApoER2/reelin signaling is able to regulate LRP3 expression, and LRP3 reduces APP protein levels, including sAPP fragments and Aβ peptide. The mechanism involved is yet to be determined, although it may be related to APP endocytosis. This study could contribute to find new strategies in aging and AD research, given that LRP3 modulation could participate in the regulation of Aβ levels.

### Limitations

The main limitation of this study is the scarce knowledge of the physiological function of LRP3 in the brain, as there are few reports about it as a neuronal receptor. We employed a well-characterized brain collection, but it would be interesting to validate our findings with an alternative collection of post-mortem cortex samples from MA individuals and cases with AD-related pathology. Despite the difference in age between non-demented and control subjects, age does not appear to be related with decreased LRP3 expression in the AD-related pathological group, but the validation of the data in age-matched groups is desirable. Development of in vivo knockouts or knockdowns of LRP3 would contribute to the understanding of the mechanism that links this receptor and APP, given that, for example, knockdown of LRP10 led to increased processing of APP to generate Aβ [48].

## Supplementary Information

**Additional file 1: Supplemental Figure 1.** Representatives whole blots. Whole blots from SH-SY5Y cell extracts, human frontal cortex from non-demented and Alzheimer’s disease subjects, and from CHO cell extracts and supernatants. The antibody employed in every blot is indicated. T = total input, B = bound fraction, U = unbound fraction, Bc: bound fraction of the negative control, Uc: bound fraction of the negative control.

**Additional file 2.** Complete microarray.

## Data Availability

All data and materials support their published claims and comply with field standards.

## Abbreviations

Aβ42: β-Amyloid protein (amino acid residues 1–42)
AD: Alzheimer’s disease
APP: Amyloid β precursor protein
ApoE: Apolipoprotein E
ApoER2/LRP8: Apolipoprotein E receptor 2
ApoER2-ICD: ApoER2 intracytoplasmic domain
ApoER2-CTF: ApoER2 C-terminal fragment
CQ: Chloroquine
LDLR: Low-density lipoprotein receptor
ND: Non-demented
VLDLR: Very low-density lipoprotein receptor
