# Supplementary figures and images for: The apolipoprotein receptor LRP3 compromises APP levels
Source: Alzheimers Res Ther. 2021 Nov 2;13:181. doi: 10.1186/s13195-021-00921-5 (PMC8565065; doi:10.1186/s13195-021-00921-5)

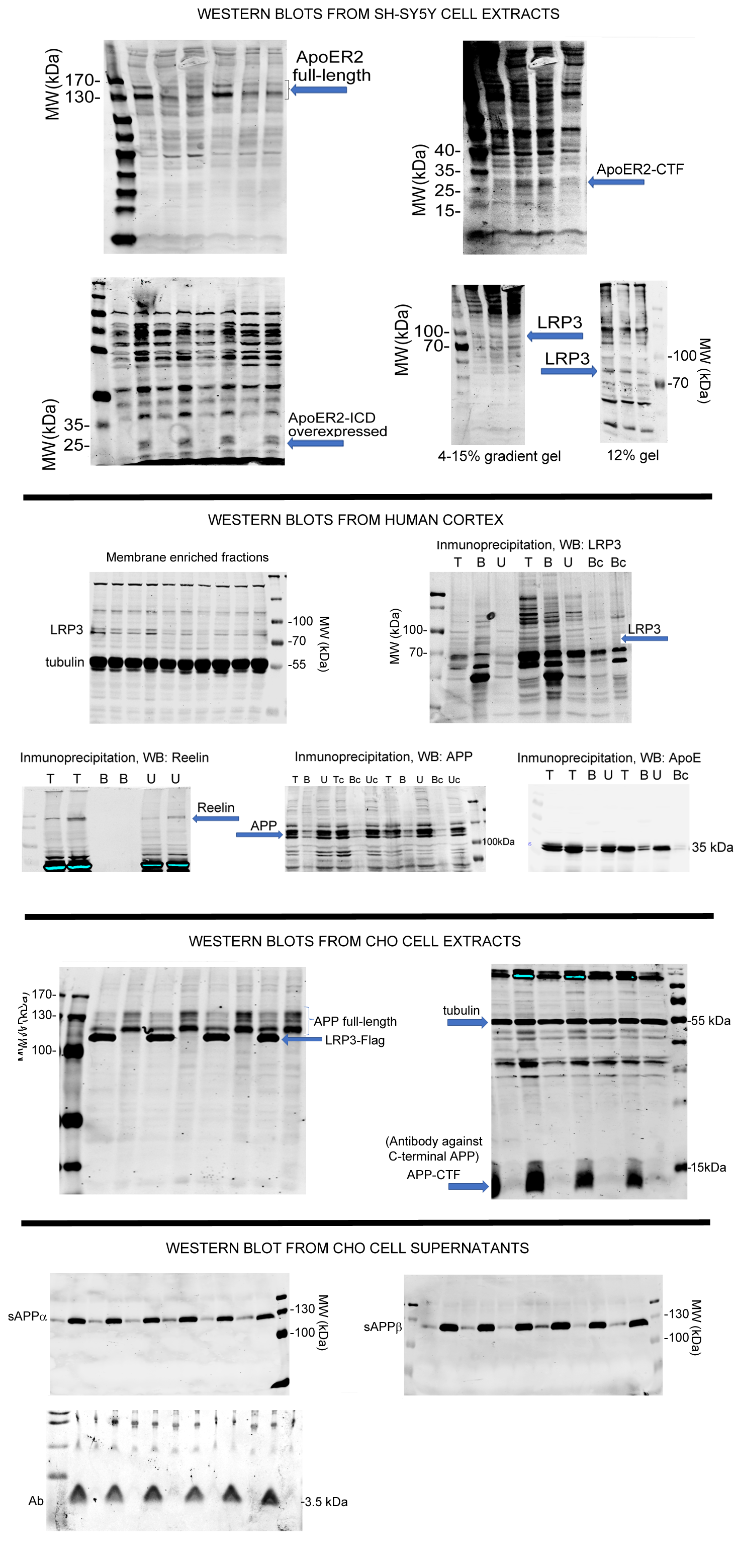

Supplement: Supplementary file 1 — Additional file 1: Supplemental Figure 1. Representatives whole blots. Whole blots from SH-SY5Y cell extracts, human frontal cortex from non-demented and Alzheimer’s disease subjects, and from CHO cell extracts and supernatants. The antibody employed in every blot is indicated. T = total input, B = bound fraction, U = unbound fraction, Bc: bound fraction of the negative control, Uc: bound fraction of the negative control. [file 13195_2021_921_MOESM1_ESM.tif]
